# Comparison of two screening tests for HIV-Associated Neurocognitive Disorder suspected Japanese patients with respect to cART usage

**DOI:** 10.1371/journal.pone.0199106

**Published:** 2018-06-14

**Authors:** Kaoru Kami-Onaga, Masao Tateyama, Takeshi Kinjo, Gretchen Parrott, Daisuke Tominaga, Ai Takahashi-Nakazato, Hideta Nakamura, Daisuke Tasato, Kyoko Miyagi, Saori Maeda, Hirotaka Arae, Hitoshi Uehara, Kazuya Miyagi, Shusaku Haranaga, Jiro Fujita

**Affiliations:** 1 Department of Infectious Diseases, Respiratory, and Digestive Medicine, Graduate School of Medicine, University of the Ryukyus, Okinawa, Japan; 2 Okinawa Study Center, The Open University of Japan, Chiba, Japan; 3 Department of Nursing, University of the Ryukyus Hospital, Okinawa, Japan; 4 Department of Hospital Pharmacy, Faculty of Medicine, University of the Ryukyus, Okinawa, Japan; Consejo Superior de Investigaciones Cientificas, SPAIN

## Abstract

In this study, we demonstrated the pervasiveness of HIV-associated neurocognitive disorders (HAND) among a selection of Japanese patients as well as evaluated and compared the Mini Mental State Examination (MMSE) and the International HIV Dementia Scale (IHDS) for use as a screening tool among combination anti-retroviral therapy (cART)-naïve and cART experienced patients. The MMSE and the IHDS have both been used as HAND screening tests around the world with variable success. It has been reported the increased usage of cART the utility of these screening tests may have been diminished due to the decreased severity of impairment and the altered pattern of neurocognitive impairments in cART era HAND patients. It is therefore possible the MMSE and the IHDS may still be useful among cART-naïve patients even in the cART era. However, only one study has investigated and compared the screening results of the IHDS among cART-naïve and cART experienced patients. All HIV positive patients who visited, or were admitted, to the Ryukyu University Hospital between January 2009 and March 2014 were evaluated for inclusion. Selected patients (n = 49) had data without omission for all tests. The overall prevalence of HAND in our cohort was 44%. The area under the curve (AUC), for all subjects using the MMSE and the IHDS, were 0.60 and 0.69, respectively. However, the AUC among cART-naïve patients were 0.58 and 0.76 for the MMSE and the IHDS, respectively. Whereas, cART experienced patients had an AUC of 0.60 and 0.61, respectively. Overall, the MMSE demonstrated a poor screening ability for HAND, regardless of cART usage (the cut-off value of 27 had a Youden's J-Index of 0.1, in all groups). Alternatively, the IHDS was moderately useful for HAND screening among cART-naïve patients (the cut-off value of 11 had a Youden's J-Index of 0.4), but performed poorly as a screening test among cART experienced patients (the cut-off value of 11 had a Youden's J-Index of 0.1).

## Introduction

HIV-associated neurocognitive disorder (HAND) is a cognitive impairment associated with HIV infection [1]. As such, HAND is an important consideration during an HIV examination since it can lead to a wide variety of challenges encountered during daily activities, such as employment, automobile driving, and medication adherence [2–4]. In patients with HIV viral load managed by combination anti-retroviral therapy (cART), it is reported that HAND has a 20–74% prevalence rate [5–7] and according to the widely used diagnostic criteria, commonly known as the Frascati criteria, a neuropsychological test battery is important for an accurate HAND diagnosis [1]. However, in order to conduct a complete neuropsychological test, it is critical to have the proper amount of time, materials, and the presence of a specialized neuropsychologist. Yet, many healthcare facilities do not have these resources. Therefore, a simple screening test, which can be conducted in any facility, to find patients with cognitive function disorder is required.

The Mini Mental State Examination (MMSE) [8] is one of the most widely used dementia screening tests. It is particularly useful for screening cortical dementia, but has also been used for HAND [9,10]. The International HIV Dementia Scale (IHDS) was published as a tool to screen patients at a high risk for HAND, without being affected by language and culture [11–13]. Both screening tests have characteristics which allow for easy implementation.

It has been previously demonstrated that the IHDS has higher sensitivity and specificity for finding HAND cases compared to the MMSE [14,15]. It is considered a useful screening tool to detect HAND cases, although its diagnostic utility in detecting mild forms of HAND is limited [12,13,16]. With the increased usage of cART, the diagnostic utility of many screening tests may have been decreased due to the diminished number of cases, reduced severity of impairment, and an altered pattern of neurocognitive impairments experienced by patients [16,17]. Indeed, the screening ability of both the MMSE and IHDS has been reportedly lower for cART experienced patients [15]. Therefore, the MMSE and IHDS are thought to be useful primarily among modern cART-naïve patients. However, only one study has investigated and compared the screening results of the IHDS among cART-naïve and cART experienced patients and showed the sensitivity of the IHDS was higher for patients receiving cART compared to cART-naïve patients [18]. This study had some limitations, for example, most patients (80/90; 89%) were taking cART and almost half of patients with cART were HIV-associated dementia (HAD), which is the most severe status of HAND. Therefore, selection bias ought to be considered and the results should be interpreted with caution.

As such, the objective here was to evaluate and compare the MMSE and the IHDS for use as a screening tool among cART-naïve and cART experienced Japanese patients. Additionally, we determine the pervasiveness of HAND among a selection of Japanese patients.

## Materials and method

### Patients

HIV patients who visited, or were admitted, to the Ryukyu University Hospital between January 2009 and March 2014, were screened for eligibility. During the same period, patients were routinely evaluated using the following neuropsychological examinations: the MMSE, IHDS, Digit Span (DS) subtest of the Wechsler adult intelligence scale-revised (WAIS-R), Trail Making Test Part A (TMT-A), Digit Symbol subtest of the WAIS-R (DST), Trail Making Test Part B (TMT-B), and the Stroop Test (ST). Patients diagnosed with: 1) neurological disorders not related to HIV infection, 2) significant traumatic brain injury, 3) infections that may affect the central nervous system, 4) current or past history of psychotic disorders, 5) current or past major depression, 6) color vision abnormality, and 7) high fever (>37.6 degrees Celsius), were excluded (n = 25).

Current CD4 count and viral load, from defined as the day closest to neuropsychological examinations (day range -34 to +4), were collected from the patient record to be used in this study. Nadir CD4 count was defined as the lowest CD4 count of all available tests, between the day of HIV infection diagnosis and the day of blood collection closest to neuropsychological examinations. Patients were further categorized into two groups for comparison, cART-naïve group and cART experienced group. The cART-naïve group was comprised of patients not treated with cART at the time neuropsychological examinations were administered. In a process concordant with national guidelines, cART was administered to these patients in a timely manner. Neuropsychological examinations, for both groups, were performed when patients were in a stable and comfortable state, in a quiet, individual room. Trained neuropsychologists administered both the screening tests and the neuropsychological battery.

### Neuropsychological test battery

We used a brief neuropsychological battery as the standard for HAND diagnosis. Attention and working memory was assessed by DS [19]. Information processing speed was assessed by TMT-A [20–23] and DST [19]. Executive function was assessed by TMT-B [20–23] and ST [23–27]. Patients, who had deteriorated by at least 1 standard deviation (SD) on two or more of the neuropsychological domains, were diagnosed as HAND. HAND patients were classified as either asymptomatic neurocognitive impairment and mild neurocognitive disorder (ANI/MND) or HAD. If patients showed a deterioration between 1–2 SD or ≥2 SD difference on two or more neuropsychological domains, they were classified as ANI/MND or HAD, respectively [1].

### Screening tests

The MMSE is an interviewer-administered questionnaire testing 5 domains (orientation, memory registration, attention and calculation, memory recall, and language), with a maximum score of 30 points [8]. A Japanese version of the MMSE, which has been widely used in Japan, was administered in our study. The phrase to measure language domain, “No ifs, ands or buts” in the original version was changed to, “Minna de chikara wo awasete tsuna wo hikimasu”; which, translated refers to the English idiom “Pull together [Join forces] and pull (the rope) together."

Alternatively, the IHDS consists of three subsets: a) timed finger tapping, which measures motor speed, b) timed alternating hand sequence, which assesses the psychomotor speed, and c) recall of four words in two minutes, which assesses memory registration and recall. Each of these subtests is rated on a scale from 0 to 4. All tests were translated and administered following previously published procedures [11]. Registration (new learning) was measured by reciting four words to the patient (dog, hat, bean, and red) using 1 second to say each of the words. The subject was asked to repeat the words, and recall the four words after the timed finger tapping, and alternating hand sequence tests were performed. Japanese translation of the words provided, “inu,” “boushi,” “mame,” and “aka” to replace the original English words.

### Statistical analysis

Statistical analysis was performed using SPSS version 21.0.0 (IBM SPSS Inc., Chicago, IL, USA) and Stata version 11.2 (Statacorp LP, College Station, TX, USA). T-test was used for age comparison. Mann-Whitney U test was used for comparing the number of education years, CD4 count, and viral load. Pearson's chi-square test was used for the comparison of categorical variables except HAD. Fisher's exact test was applied to HAD because expected number of patients in either group was less than 5. Using the results from the neuropsychological test battery as a gold standard for HAND diagnosis, a Receiver Operator Characteristic (ROC) curve was generated to evaluate the screening accuracy of the MMSE and IHDS for all subjects, and the subsets of patients categorized as pre-cART or cART group. Youden's J-Index (J) was calculated manually.

### Ethics

The Institutional Ethics Committees of the University of the Ryukyus approved this study (H26-256). The need for informed consent from each patient for inclusion was waived because this study was retrospective in approach, which caused no additional adverse events in any subject.

## Results

**Fig 1:**
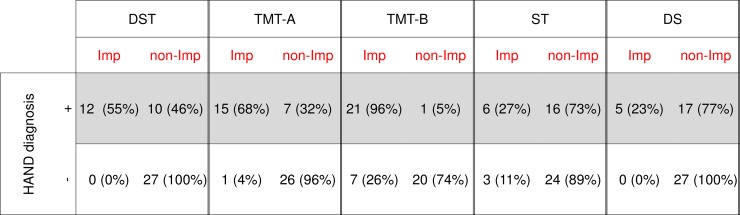
Patient scores for each neurophychological test for HAND diagnosis (n = 49). Impairment (Imp) was considered as mental deterioration of at least 1 standard deviation. Non-impairment (non-Imp) patients were considered as having minimal mental deterioration ranging from less than 1 standard deviation to normal cognitive abilities. Abbreviations: DST: Digit Symbol Test, TMT-A; Trail Making Test Part A, TMT-B; Trail Making Test Part B, ST; Stroop Test, DS; Digit Span.

During the study period, 49 patients meeting our criteria were identified. The patient characteristics are listed in Table 1. At the time of neuropsychological examinations, 27 patients had not received cART (cART-naïve group) and 22 patients were currently receiving cART (cART experienced group). An official diagnosis of HAND was revealed for 44% of patients, including 15 cART-naïve and 7 cART experienced patients. There were no significant differences in age, gender, education years, number of HAND diagnoses, and nadir CD4 counts between the cART-naïve and cART experienced group. The current CD4 counts for cART-naïve patients were significantly lower than those of cART experienced patients (p<0.001). Conversely, current viral load of the cART-naïve group was significantly higher than that of cART experienced group (p<0.001). The ratio of ANI/MND and HAD patients was 12:3 and 7:0 among the cART-naïve and cART experienced groups, respectively. Only three cART-naïve patients (6% of total cohort) were diagnosed with HAD. As shown in Fig 1, TMT-B was the most sensitive test for the detection of cognitive impairment and 96% of HAND patients showed mental deterioration of at least 1 SD on TMT-B, followed by TMT-A (68%), DST (55%), ST (27%), and DS (23%).

**Table 1:** Patient background and laboratory findings.

Variable	Total (n = 49)	cART-naïve (n = 27)	cART experienced (n = 22)	P-value
Age (years)*	42.0 (9.5)	40.3 (10.5)	44.1 (7.8)	0.17
Male gender (%)	46 (93.9)	24 (88.9)	22 (100)	0.11
Education (years)^†^	14 (9–18)	14.0 (9–18)	14.0 (9–16)	0.74
Nadir CD4 count (cells/μl)^†^	62.0 (3–778)	57.0 (3–778)	73.0 (4–263)	0.67
Current CD4 count (cells/μl)^†^	335 (4–1256)	80 (4–968)	616.5 (254–1256)	<0.001
Current VL (log10 copies/ml)^†^	0.9 (0–6.6)	5.2 (0–6.6)	0 (0–0.9)	<0.001
HAND (%)	22 (44.9)	15 (55.6)	7 (31.8)	0.10
ANI/MND (%)	19 (38.8)	12 (44.4)	7 (31.8)	0.37
HAD (%)	3 (6.1)	3 (11.1)	0 (0)	0.11
Duration of cART (month)*	_	_	97.2 (42.9)	_

*mean(±SD)

^†^median (range)

Abbreviations: cART; combination anti-retroviral treatment, VL; viral load, HAND; HIV-associated neurocognitive disorder, ANI/MND; asymptomatic neurocognitive impairment and mild neurocognitive disorder, HAD; HIV-associated dementia.

**Fig 2:**
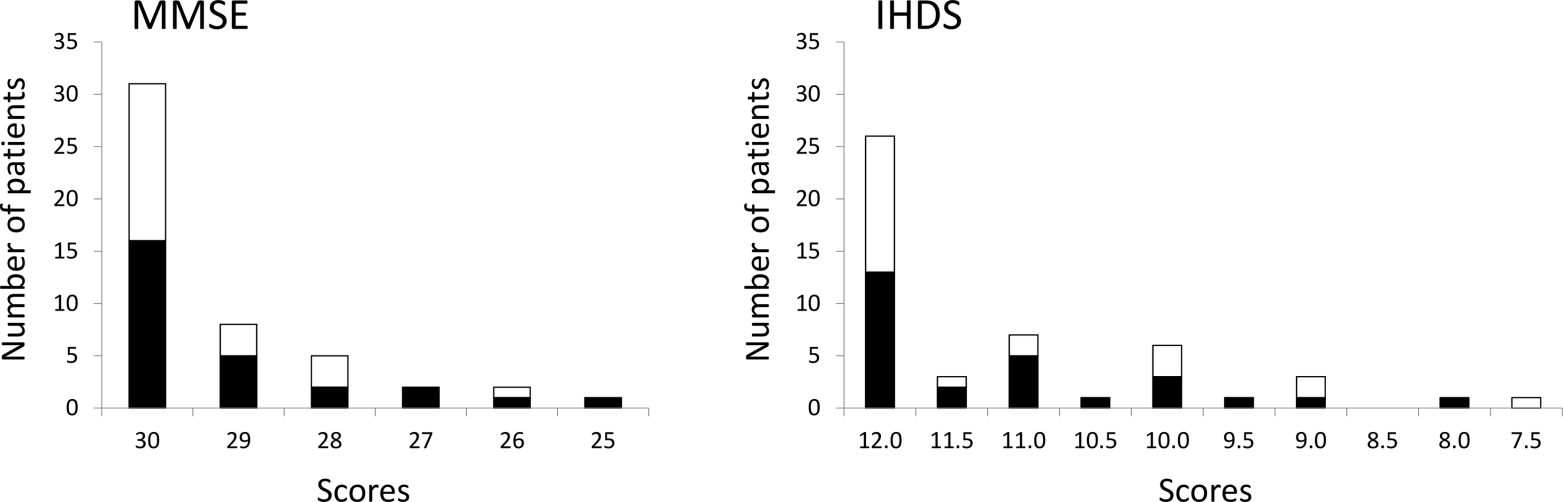
Patient score distribution for MMSE and IHDS (n = 49). Black and white bar indicate cART-naive and cART experienced patients, respectively.

**Fig 3:**
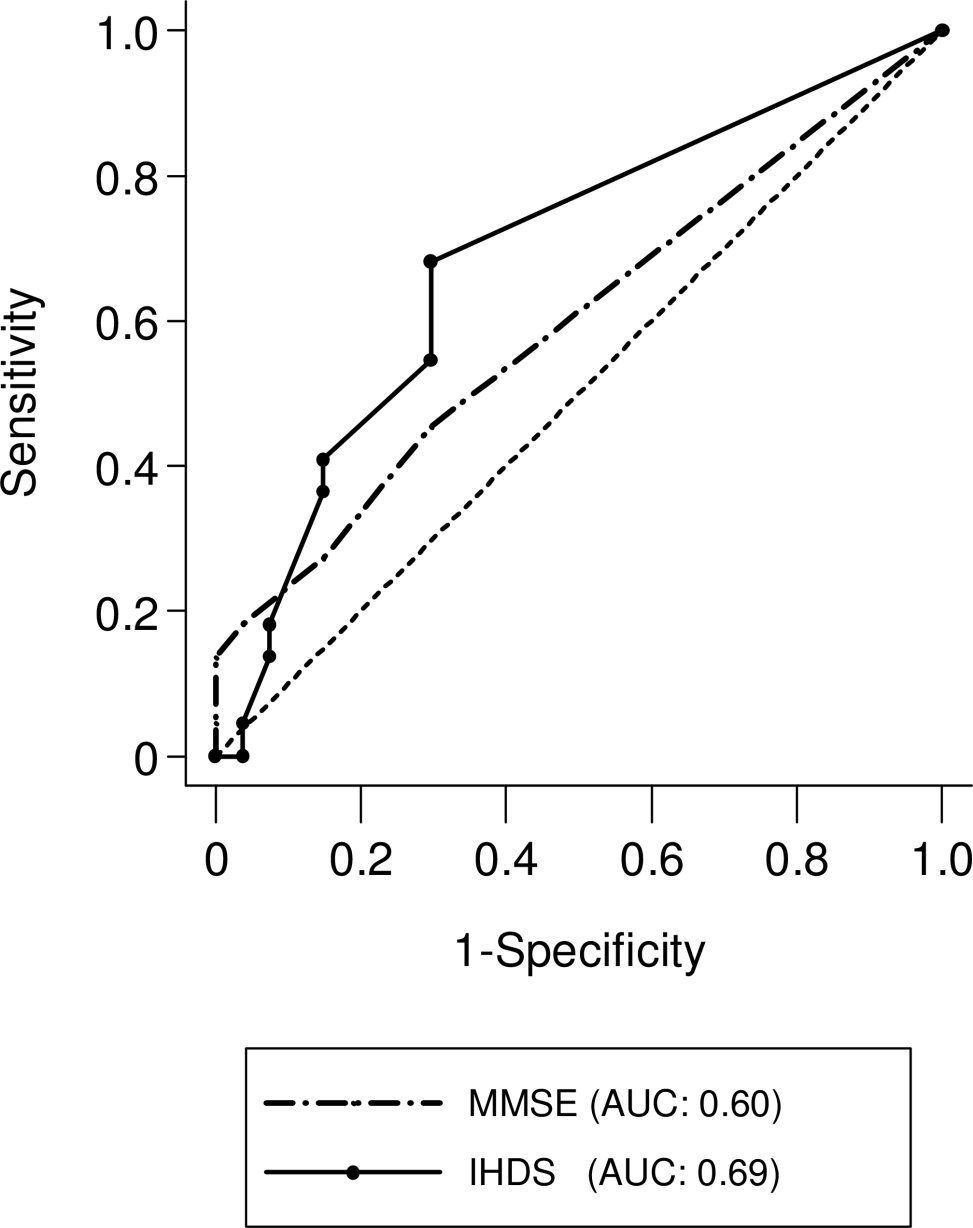
ROC curve of MMSE and IHDS among all subjects (n = 49). Receiver Operator Characteristic (ROC) curve generated by using the results from the neuropsychological test battery as a gold standard for HAND diagnosis.

**Fig 4:**
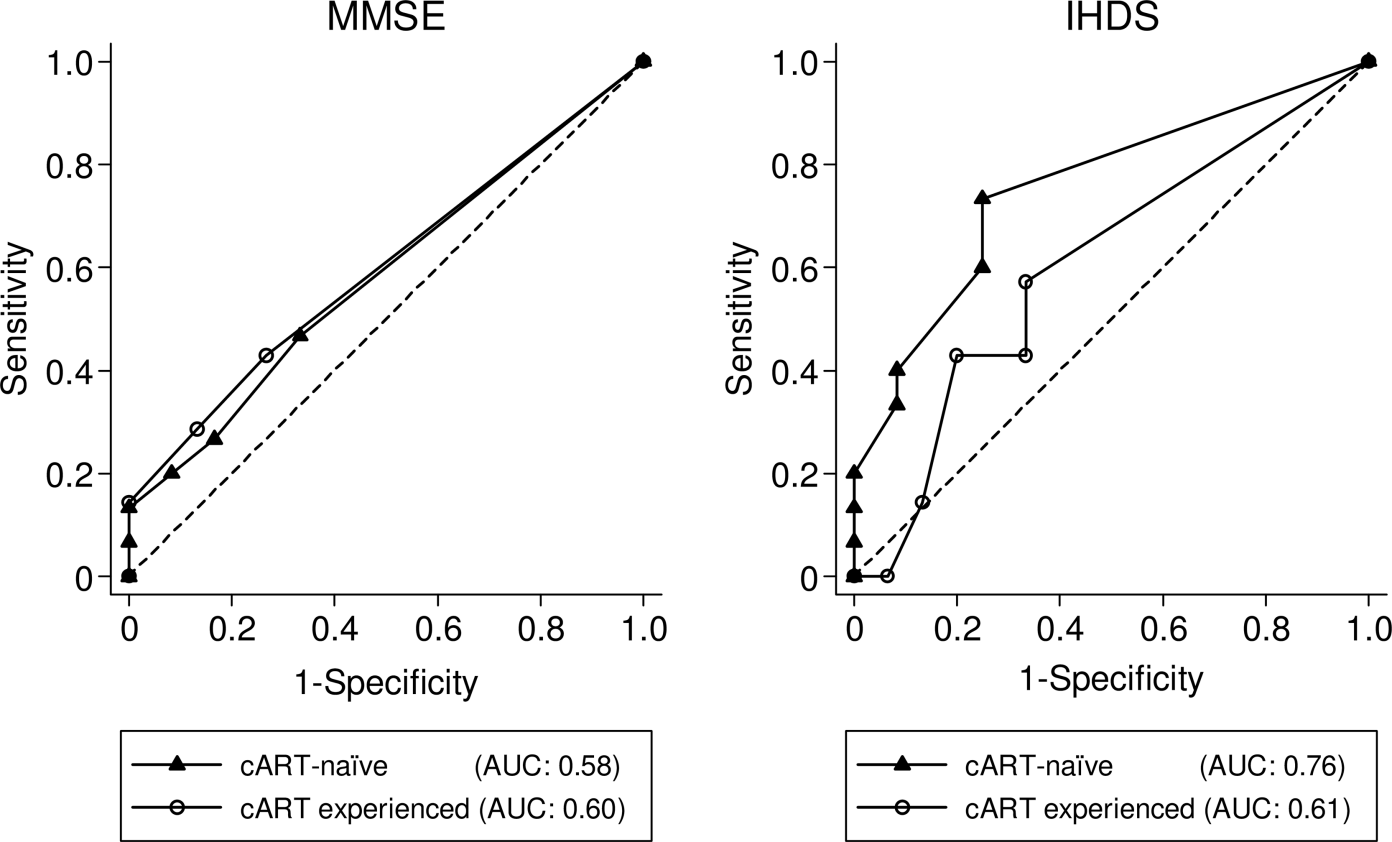
ROC curve of MMSE and IHDS among cART-naïve (n = 27) and cART experienced patients (n = 22). Receiver Operator Characteristic (ROC) curve for cART-naïve and cART experienced patients generated by using the results from the neuropsychological test battery as a gold standard for HAND diagnosis.

Overall, 63% of patients scored 30 on the MMSE and 53% scored 12 on the IHDS. The cART-naive patients had an average score of 29.1 for the MMSE and 11.1 for the IHDS, and cART experienced patients had 29.4 and 11.1 for the average, respectively (Fig 2). As shown in Fig 3, the AUC for the MMSE and IHDS in all subjects were 0.60 and 0.69, respectively. Dividing the cohort into cART-naïve and cART experienced groups, altered the AUCs. The MMSE and IHDS AUC among cART-naïve patients were 0.58 and 0.76, but were 0.60 and 0.61 for cART experienced patients, respectively (Fig 4). Out of 22 patients diagnosed with HAND, only 4 patients were correctly screened using the MMSE (cut-off value of ≤27 [15]), however, 12 patients were correctly screened using the IHDS (cut-off value of ≤11 [15,18]). The sensitivity and specificity of the MMSE at a cut-off value of ≤27 for all subjects were 18% and 96%, respectively (J = 0.1). In Table 2, the sensitivity of the MMSE in cART-naïve patients was shown to be slightly increased compared to the cART experienced group (cART-naïve; 20%, cART; 14%). Conversely, the specificity of the MMSE among cART-naïve group was lower than that of the cART experienced group (cART-naïve; 92%, cART; 100%). However Youden's J-Index remained unchanged at 0.1 for each subset. On the other hand, using a cut-off value of ≤11 for the IHDS, the sensitivity and specificity among all patients were 55% and 70%, respectively (J = 0.3). When divided, sensitivity and specificity were 60% and 75% for cART-naïve, and 43% and 67% for cART experienced patients, respectively (Table 3). The Youden’s J-Index for diagnostic accuracy among each subset was 0.4 for cART-naïve and 0.1 for cART experienced patients.

**Table 2:** Diagnostic accuracy of MMSE between cART-naïve and cART experienced patients.

		MMSE			
		≤26	≤27	≤28	≤29
All participants (n = 49)	Number identified as impaired	3	5	10	18
	Sensitivity (%)	14	18	27	45
	Specificity (%)	100	96	85	70
	PPV (%)	100	80	60	56
	NPV (%)	59	59	59	61
cART-naïve (n = 27)	Number identified as impaired	2	4	6	11
	Sensitivity (%)	13	20	27	47
	Specificity (%)	100	92	83	67
	PPV (%)	100	75	67	64
	NPV (%)	48	48	48	50
cART experienced (n = 22)	Number identified as impaired	1	1	4	22
	Sensitivity (%)	14	14	29	43
	Specificity (%)	100	100	87	73
	PPV (%)	100	100	50	43
	NPV (%)	71	71	72	73

Abbreviations: MMSE; Mini Mental State Examination, cART; combination anti-retroviral treatment, PPV; positive predictive value, NPV; negative predictive value.

**Table 3:** Diagnostic accuracy of IHDS between cART-naïve and cART experienced patients.

		IHDS			
		≤10	≤10.5	≤11	≤11.5
All participants (n = 49)	Number identified as impaired	12	13	20	23
	Sensitivity (%)	36	41	55	68
	Specificity (%)	85	85	70	70
	PPV (%)	67	69	60	65
	NPV (%)	62	64	66	73
cART-naïve (n = 27)	Number identified as impaired	6	7	12	14
	Sensitivity (%)	33	40	60	73
	Specificity (%)	92	92	75	75
	PPV (%)	83	86	75	79
	NPV (%)	52	55	60	69
cART experienced (n = 22)	Number identified as impaired	6	6	8	9
	Sensitivity (%)	43	43	43	57
	Specificity (%)	80	80	67	67
	PPV (%)	50	50	38	44
	NPV (%)	75	75	71	77

Abbreviations: IHDS; International HIV Dementia Scale, cART; combination anti-retroviral treatment, PPV; positive predictive value, NPV; negative predictive value.

## Discussion

Multiple studies suggest that due to the lack of an internationally standardized and endorsed screening tool for HAND, local assessment of screening tools is necessary [18,28], Our study is the first attempt to validate and compare HAND screening tools, with respect to cART usage, in a homogenous Japanese demographic. The comparison of the MMSE and IHDS performance between roughly the same number of cART-naïve and cART experienced patients provides additional value to Japanese HIV-infected patients, many of whom may not receive an HIV diagnosis until several years after infection [29]. Overall, an official diagnosis of HAND was revealed for 44% of this cohort.

The primary aim of this study investigated the ability of the MMSE and IHDS to screen for HAND in both cART-naïve and cART experienced patients, in an effort to elucidate the impact cART has on the reliability of screening tests for HAND suspected patients. The MMSE was confirmed to be insufficiently sensitive as a screening tool for HAND across all patients, and cART experienced patients did not perform differently than cART-naïve patients. Multiple studies have shown the MMSE is a poor screening tool for HAND, however these studies either do not analyze results with respect to cART usage [30], or do not distinguish between those treated and those not [15,30–32]. Although, Power et al., has investigated the performance of the MMSE in the pre-cART era [30], a recent evaluation of the MMSE performance among cART-naïve patients remained unknown. This study revealed, the MMSE is equally inadequate as a screening tool for both cART-naïve and cART patients. It is possible the MMSE is unable to capture the diminished mental recognition in patients with HAND. HAND causes a subcortical dementia and the MMSE is most useful in cortical dementia [33,34]. Our data agrees with previously published data demonstrating the MMSE is not recommended as a screening tool for HAND in HIV patients [15,31,32].

In contrast, the IHDS at a cut-off value of ≤11 (J = 0.3) was determined to be a more sensitive screening tool for HAND than the MMSE at a cut-off value of ≤27 (J = 0.1), but with less specificity. For patients receiving cART, sensitivity and specificity of the IHDS at a cut-off value of ≤11 were reduced (J = 0.1), demonstrating that cART usage can potentially alter the pattern of HAND associated impairment and thus alter the reliability of the IHDS. Currently, no common ground on the performance of the IHDS for HAND screening has been established [11, 32,35–37]. One study reports the IHDS performs poorly as a screening tool for HAND in patients receiving cART [15]. Another has shown the IHDS to be a reliable tool for HAND screening in patients not receiving cART [38]. A third claims that although no current tool is adequate in screening for any HAND, a combination of the IHDS and an individually designed rapid assessment tool provided a good screening alternative [28]. However, the study designs of the previous reports are heterogeneous, with prominent differences being: inclusion criteria used, characteristics and risk factors analyzed, comparisons performed by disease severity, number and style of neuropsychological exams, cut-off values used, and study size. As a result, many studies were unable compare the performance of the IHDS between cART-naïve and cART experienced patients adequately. For example, Marin-Webb, et al. compared the accuracy of the IHDS for patients taking and not taking cART, and demonstrated the sensitivity of the IHDS was higher for patients receiving cART compared to cART-naïve patients [18]. However, this relationship was reversed when comparing specificity. Marin-Webb explained this difference was due to selection bias, the difference in the number of patients for each group was significant, and thus the results should be interpreted with caution.

As recently as 2013, the IHDS was considered a useful tool for screening HAND [13]. In this study, we demonstrate the IHDS was most accurate for cART-naïve patients. Both the sensitivity and specificity of the IHDS were decreased in patients receiving cART. It is well known that cART leads to a drastic decrease in the number of HAD cases [5,39], as demonstrated in this cohort. Within the cART experienced group, no patients were diagnosed with HAD. However, 11% (n = 3) of cART-naïve patients were diagnosed with HAD. Indeed, it has been reported that IHDS is especially useful for HAD patients, whereas its ability to find cases of ANI/MND is poor [36]. Although our data shows no significant difference in the number of HAD cases for each treatment group (Table 1), we investigated the IHDS' reliability with HAD cases removed from the data. Supplementary Figures (S1 Fig, S2 Fig and S3 Fig) continues to show the IHDS as more sensitive and specific for cART-naïve patients (J = 0.3) than cART experienced patients (J = 0.1). However, the difference may also be due to the gradation of HAND severity in each of the subset groups.

Differences may also exist in the domain or number of the cognitive ability impairments between cART-naïve and cART experienced patients. Cognitive decline regions of HAND patients diagnosed in the pre-cART era included a decrease in motor function, language fluency, and cognitive information processing speed. However, HAND patients are now documented to experience a decline in learning and executive function [17]. Since the cognitive functions evaluated by the IHDS include motor function, psychomotor speed and recall [11], it is possible the decreased learning and executive function, seen in cART experienced patients, cannot be measured.

Even with its relatively low burden of HIV positive patients, Japan has made long strides in addressing HAND, however there remains no nationally recognized screening test. In that sense, this manuscript lends itself to establishing baseline results. Multiple countries have also performed research regarding the IHDS in an effort to make screening for HAND more accurate [18,28]. While this research has some limitations, it remains novel and necessary due to the lack of data from the Japanese population.

This investigation was conducted within a single center and has a relatively small number of subjects; as such its investigative power may be low. In addition, our study population includes mostly men, which is representative of the epidemiology in Japan [40]. Although many studies have shown that women are at higher risk for cognitive impairment than men [16], no significant differences between the presented data and an all male subset of the original cohort used in the study (S1 Table, S4 Fig, S5 Fig and S6 Fig).

Furthermore, although we referred to the Frascati criteria [1] in formulating our test battery, the resulting battery could only include the health data common among Japanese people. Currently, there is no standardized national data for executive function and motor skills for the Japanese HIV-negative population [9], and without this normative data the conversion to standard deviations is impossible and a subtest in that area is ultimately useless. Therefore, some tests suggested by the Frascati criteria, were not feasible and our “gold standard” neuropsychological exam is not exactly as recommended by the Frascati criteria. Nevertheless, this study should be considered as a foundation for future studies as national standards become established and newer screening tests are developed. Lastly, confounding factors such as current employment, duration of HIV infection, patient specific cART regimens, and length of cART were not investigated, but may influence our results. However, the Japanese population has already been established as particularly suitable for investigating the relationship between cART and HAND as they have a relatively uniform genetic and cultural background [29].

In conclusion, our data suggested the MMSE should not be recommended as a screening tool for HAND among Japanese people. Alternatively, the IHDS may prove useful among patients suspected of HAND who have not yet initiated cART. Further research is needed to ensure our data is generalizable to either a larger Japanese population or other populations worldwide.

## Supporting information

S1 Dataset Raw data. (XLSX)

S1 Table Patient background and laboratory findings in men. Abbreviations: cART; combination anti-retroviral treatment, VL; viral load, HAND; HIV-associated neurocognitive disorder, ANI/MND; asymptomatic neurocognitive impairment and mild neurocognitive disorder, HAD; HIV-associated dementia.
(DOCX)

S1 Fig ROC curve of MMSE and IHDS among all ANI/MND patients (n = 46). Receiver Operator Characteristic (ROC) curve for all ANI/MND patients generated by using the results from the neuropsychological test battery as a gold standard for HAND diagnosis.
(DOCX)

S2 Fig ROC curve of MMSE and IHDS among cART-naïve patients with ANI/MND (n = 24). Receiver Operator Characteristic (ROC) curve for cART-naïve patients with ANI/MND generated by using the results from the neuropsychological test battery as a gold standard for HAND diagnosis.
(DOCX)

S3 Fig ROC curve of MMSE and IHDS among cART-experienced patients with ANI/MND (n = 22). Receiver Operator Characteristic (ROC) curve for cART-experienced patients with ANI/MND generated by using the results from the neuropsychological test battery as a gold standard for HAND diagnosis.
(DOCX)

S4 Fig ROC curve of MMSE and IHDS among all male patients (n = 46). Receiver Operator Characteristic (ROC) curve for all male patients generated by using the results from the neuropsychological test battery as a gold standard for HAND diagnosis.
(DOCX)

S5 Fig ROC curve of MMSE and IHDS among cART-naïve male patients (n = 24). Receiver Operator Characteristic (ROC) curve for cART-naïve male patients generated by using the results from the neuropsychological test battery as a gold standard for HAND diagnosis.
(DOCX)

S6 Fig ROC curve of MMSE and IHDS among cART-experienced male patients (n = 22). Receiver Operator Characteristic (ROC) curve for cART-experienced male patients generated by using the results from the neuropsychological test battery as a gold standard for HAND diagnosis.
(DOCX)
