# Supplementary material for: Comparison of two screening tests for HIV-Associated Neurocognitive Disorder suspected Japanese patients with respect to cART usage
Source: PLoS One. 2018 Jun 14;13(6):e0199106. doi: 10.1371/journal.pone.0199106 (PMC6002083; doi:10.1371/journal.pone.0199106)
Supplement: S1 Table — Abbreviations: cART; combination anti-retroviral treatment, VL; viral load, HAND; HIV-associated neurocognitive disorder, ANI/MND; asymptomatic neurocognitive impairment and mild neurocognitive disorder, HAD; HIV-associated dementia. (DOCX) [file pone.0199106.s002.docx]

| Variable | Total (n=46) | cART-naïve | cART-experienced | P-value |
| --- | --- | --- | --- | --- |
|  |  | (n=24) | (n=22) |  |
| Age (years)* | 41.9 (9.5） | 40 (10.6) | 44.1 (7.8) | 0.14 |
| Male gender (%) | 46 (100) | 24 (100) | 22 (100) |  |
| Education (years)^†^ | 14 (9-18) | 14.0 (9-18) | 14.0 (9-16) | 0.6 |
| Nadir CD4 count (cells/μl)^†^ | 64.5 (3-778) | 62.0 (3-778) | 73.0 (4-263) | 0.8 |
| Current CD4 count (cells/μl)^†^ | 342.5 (4-1256) | 82.0 (4-968） | 616.5 (254-1256） | <0.001 |
| Current VL (log10 copies/ml)^†^ | 0 (0-6.6) | 5.2 (0-6.6) | 0 (0-0.9) | <0.001 |
| HAND (%) | 22 (47.8) | 15 (62.5) | 7 (31.8) | 0.04 |
| ANI/MND (%) | 19 (41.3) | 12 (50) | 7(31.8) | 0.21 |
| HAD (%) | 3 (6.5) | 3 (12.5) | 0(0) | 0.09 |
| Duration of cART (month)* | _ | _ | 97.2 (42.9) | _ |

**S1 Table. Patient background and laboratory findings in men.**

*mean(±SD), ^†^median (range)

Abbreviations: cART; combination anti-retroviral treatment, VL; viral load, HAND; HIV-associated neurocognitive disorder, ANI/MND; asymptomatic neurocognitive impairment and mild neurocognitive disorder, HAD; HIV-associated dementia.
