# Supplementary figures and images for: Comparison of two screening tests for HIV-Associated Neurocognitive Disorder suspected Japanese patients with respect to cART usage
Source: PLoS One. 2018 Jun 14;13(6):e0199106. doi: 10.1371/journal.pone.0199106 (PMC6002083; doi:10.1371/journal.pone.0199106)

**S1 Fig. ROC curve of MMSE and IHDS among all ANI/MND patients (n=46).**


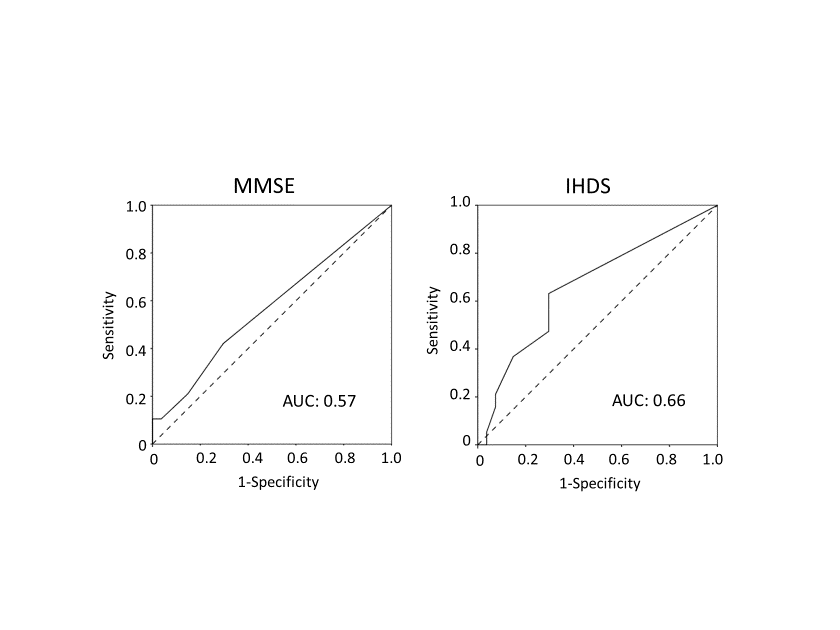

Supplement: S1 Fig — Receiver Operator Characteristic (ROC) curve for all ANI/MND patients generated by using the results from the neuropsychological test battery as a gold standard for HAND diagnosis. (DOCX) [file pone.0199106.s003.docx]

**S2 Fig. ROC curve of MMSE and IHDS among cART-naïve patients with ANI/MND (n=24).**


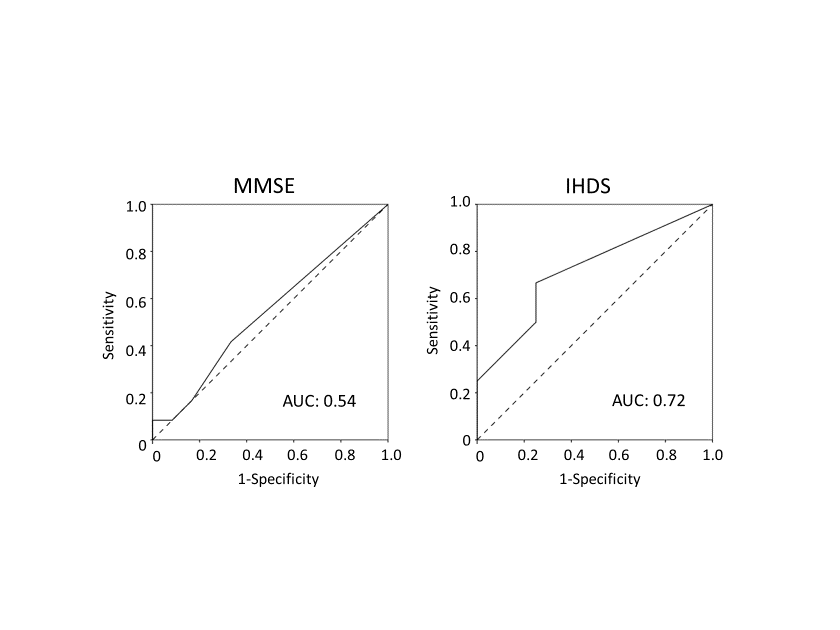

Supplement: S2 Fig — Receiver Operator Characteristic (ROC) curve for cART-naïve patients with ANI/MND generated by using the results from the neuropsychological test battery as a gold standard for HAND diagnosis. (DOCX) [file pone.0199106.s004.docx]

**S3 Fig. ROC curve of MMSE and IHDS among cART-experienced patients with ANI/MND (n=22).**

**
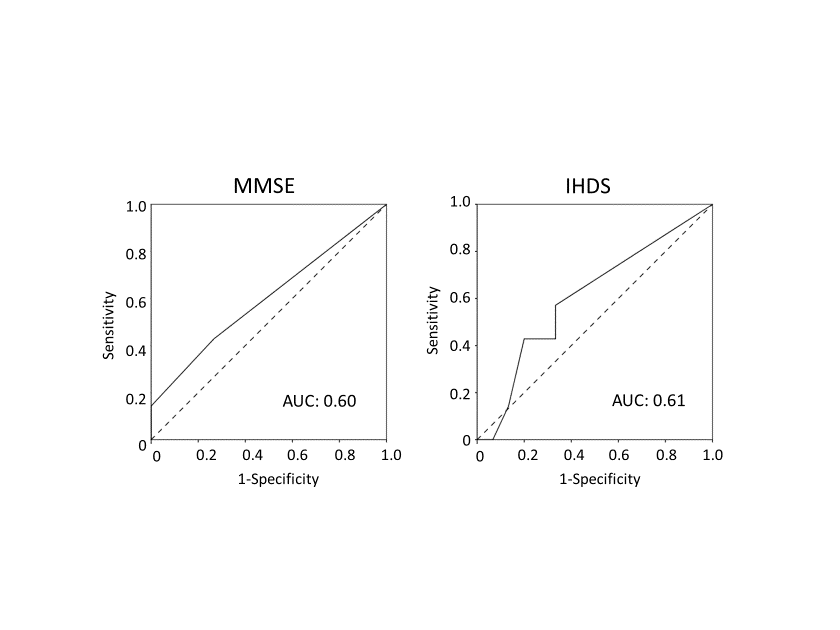
**

Supplement: S3 Fig — Receiver Operator Characteristic (ROC) curve for cART-experienced patients with ANI/MND generated by using the results from the neuropsychological test battery as a gold standard for HAND diagnosis. (DOCX) [file pone.0199106.s005.docx]

**S4 Fig. ROC curve of MMSE and IHDS among all male patients (n=46).**

**
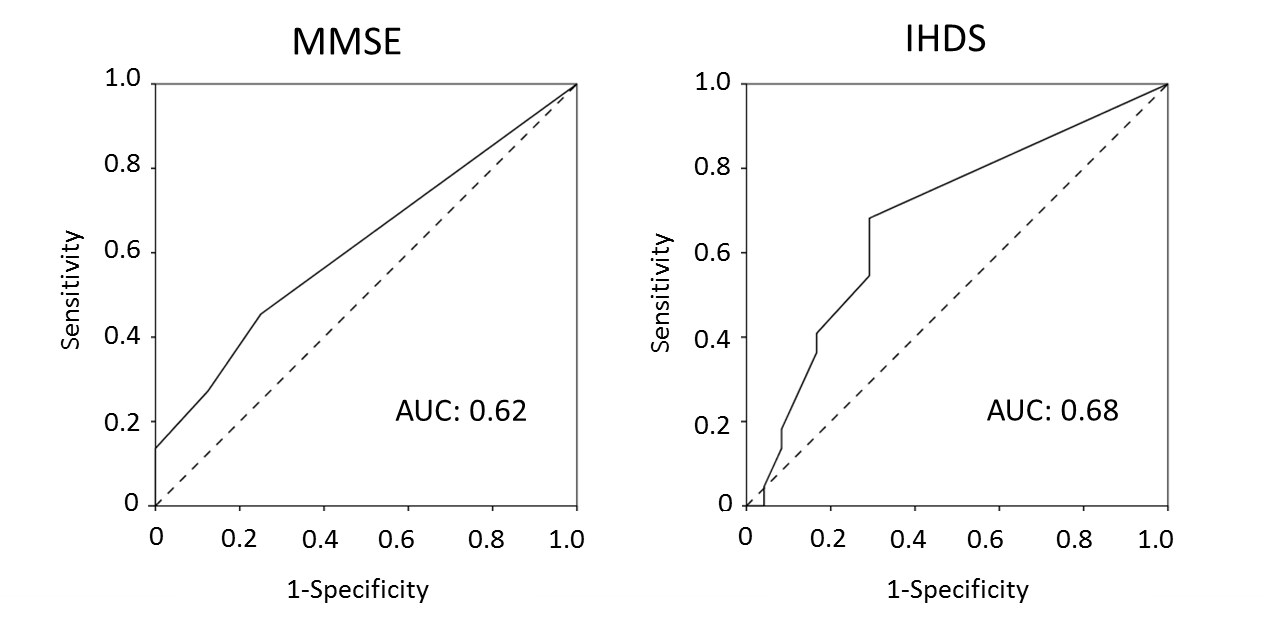
**

Supplement: S4 Fig — Receiver Operator Characteristic (ROC) curve for all male patients generated by using the results from the neuropsychological test battery as a gold standard for HAND diagnosis. (DOCX) [file pone.0199106.s006.docx]

**S5 Fig. ROC curve of MMSE and IHDS among cART-naïve male patients (n=24).**

**
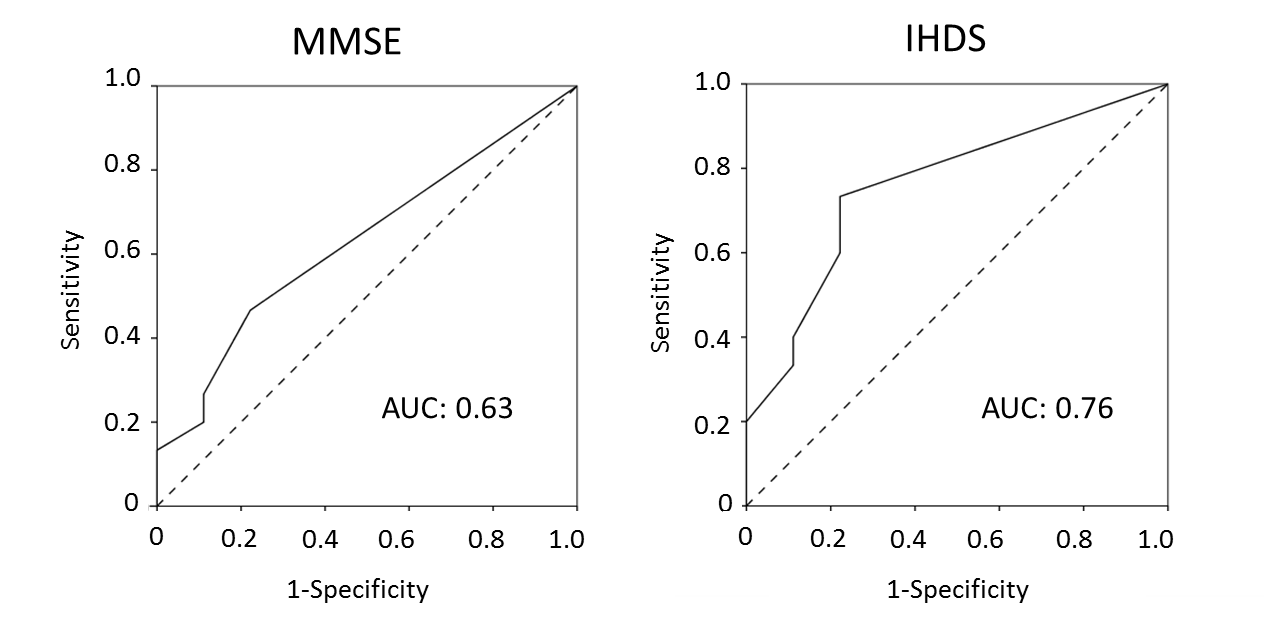
**

Supplement: S5 Fig — Receiver Operator Characteristic (ROC) curve for cART-naïve male patients generated by using the results from the neuropsychological test battery as a gold standard for HAND diagnosis. (DOCX) [file pone.0199106.s007.docx]

**S6 Fig. ROC curve of MMSE and IHDS among cART-experienced male patients (n=22).**

**
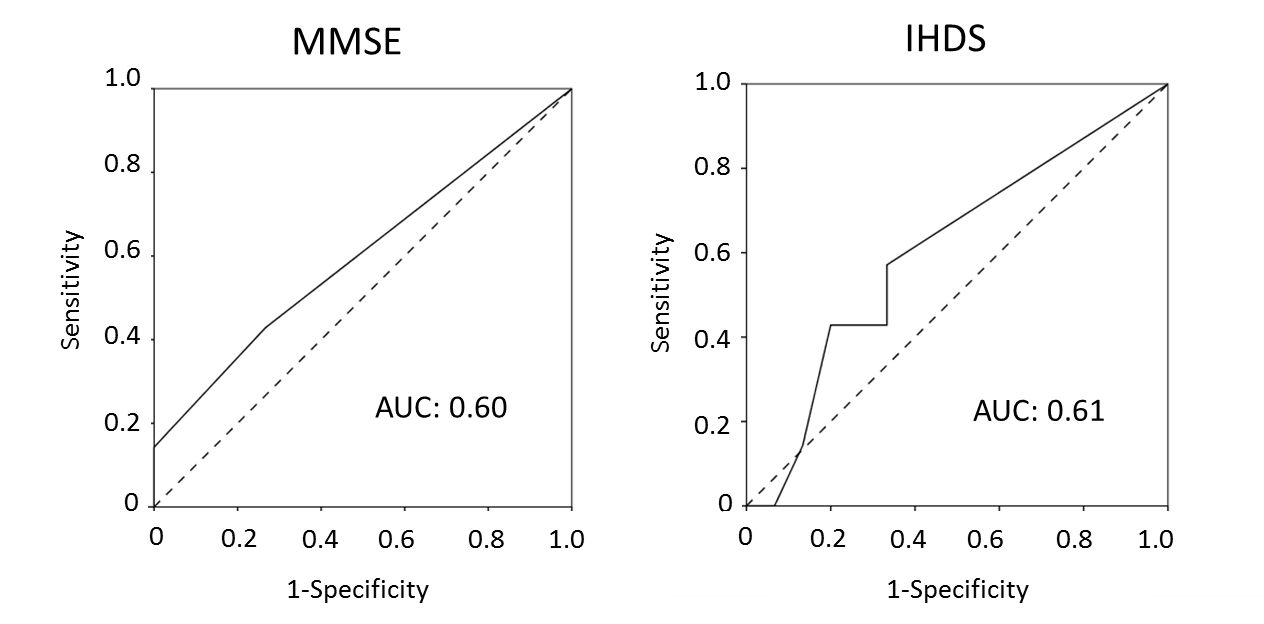
**

Supplement: S6 Fig — Receiver Operator Characteristic (ROC) curve for cART-experienced male patients generated by using the results from the neuropsychological test battery as a gold standard for HAND diagnosis. (DOCX) [file pone.0199106.s008.docx]
